# Moderate Recurrent Hypoglycemia Markedly Impairs Set-Shifting Ability in a Rodent Model: Cognitive and Neurochemical Effects

**DOI:** 10.2174/1876524601205010001

**Published:** 2012

**Authors:** Vaishali Jahagirdar, Justin Ramcharitar, Victoria E. Cotero, Ewan C. McNay

**Affiliations:** 1 Behavioral Neuroscience and Center for Neuroscience Research, University at Albany, Albany, NY 12222, USA; 2 GE Global Research, USA; 3 Excelsior College, Albany, NY 12203, USA

**Keywords:** Dopamine, glucose, medial prefrontal cortex, set-shifting ability, recurrent hypoglycemia

## Abstract

Recurrent hypoglycemia (RH) is the major complication of intensive insulin treatment for diabetes mellitus. Of particular concern is the perceived potential for long-term impact of RH on cognition. Because diabetic patients have been reported to have deficits in mental flexibility and judgment, both generally considered to be mediated predominantly by the prefrontal cortex, the purpose of the present study was to determine whether RH would affect prefrontal cortex function. Medial prefrontal cortex (mPFC)-mediated set-shifting ability was tested in male Sprague-Dawley rats using a maze-based, food-reward Set-Shift task analogous to the Wisconsin card-sorting task. The performance measure was the number of trials to criterion on both day 1 (initial rule-learning) and day 2 (set-shifting in response to a changed contingency). *In vivo* microdialysis was used to measure mPFC extracellular glucose, lactate, pyruvate, glutamate, and dopamine. Post-mortem measures within the mPFC included glucose transporter 3 (GluT3) and c-Fos. RH animals had enhanced performance on day 1, consistent with previous work that showed RH to improve subsequent hippocampal function when euglycemic. The key finding of the present work is that RH led to impaired set-shifting performance on day 2, suggesting impairment in e.g. mental flexibility. Consistent with this finding, RH animals show decreased mPFC glycolysis on day 2 compared to controls. Our data show that RH can lead to subsequent impaired judgment, accompanied by reduced prefrontal cortex function. The findings suggest a potential underlying mechanism for the impaired judgment seen in diabetic patients.

## INTRODUCTION

Intensive insulin therapy to prevent hyperglycemia is the current recommended gold standard for treatment of type 1 diabetes (T1DM), based on the results of the landmark Diabetes Control and Complications Trial and other studies; insulin treatment is also a common therapy for type 2 diabetic patients [1–3]. One major drawback of intensive insulin therapy is the increased risk of recurrent hypoglycemia (RH) [4–6]. RH leads to breakdown of autonomic counter-regulatory responses (commonly termed hypoglycemia-associated autonomic failure, or HAAF) and subsequent hypoglycemia unawareness [7, 8]. Patients often have significant fear of the long-term neural and cognitive consequences of potential interruptions in glucose supply to the brain which reduces compliance with therapy [7–9]. The long term cognitive and neural consequences of RH remain uncertain, not least because of the difficulty in human studies of accurately parsing the effects of RH from such confounds as duration of diabetes, age of onset, hyperglycemic neuropathies, and so on [10–13].

The first studies of RH in animal models, focusing on the impact of RH on hippocampal function, showed that RH can indeed affect not only subsequent cognitive performance but also, for example, brain metabolism and synaptic plasticity [14, 15, reviewed in 16]. Specifically, it has been previously shown that spatial memory performance was enhanced by prior RH if tested at euglycemia, but impaired if tested during further hypoglycemia; a matching pattern of changes was seen in hippocampal glucose transport and metabolism, and in synaptic plasticity [14]. The finding that c-Fos activity, a marker of neural activation, is increased by acute hypoglycemia but diminishes subsequent to RH, further suggests that RH affects subsequent neural function [17].

Clinically, a history of RH and impaired awareness of hypoglycemia is associated with impaired judgment and decision-making, domains mediated in large part by the prefrontal cortex [18, 19]. When a cohort of T1DM patients was asked to estimate their blood glucose levels and then asked whether they would drive, 38% decided to drive despite blood glucose levels below 40 mg/dl [20]. A second study found that 43% of T1DM patients with impaired awareness of hypoglycemia decided to drive despite hypoglycemia [21]. Judgment and decision-making are both regulated by executive processes that appear to be primarily localized to the medial prefrontal cortex (mPFC) [18, 19, 22–24]. Manipulations of receptors for dopamine, a primary neurotransmitter within the mPFC, modulate mental flexibility [25–28]; however, neither mPFC function nor dopaminergic activity have been examined as potential mechanistic links between RH and impaired decision-making.

We examined the impact of recurrent hypoglycemia on mPFC-mediated behaviors. We used a rodent cognitive task that relies on ability to perform cognitive shifts and is analogous to human Wisconsin card-sorting test [29, 30]. The Set-Shift task engages higher-order functions such as, attentional processes, working memory, and decision-making ability and is detailed in the Methods section [22, 23, 31]. In addition to behavioral measures, we used *in vivo* microdialysis of the mPFC before, during, and after behavioral testing as our previous studies of RH suggested that in addition to cognitive changes, we might see alterations in neural metabolism during task performance. Post mortem, we additionally measured within mPFC protein expression of c-Fos and neuronal glucose transporter 3 (GluT3).

## MATERIALS AND METHODS

### Animals

All procedures were approved by the Institutional Animal Care and Use Committee and were in accordance with NIH mandated principles of laboratory animal care. During one week of acclimatization, animals were housed in pairs in a temperature-and light-controlled room (12-h light, 12-h dark; lights on at 08:00 AM) with food and water available *ad libitum.* After this, from the 3^rd^ day of handling and during the habituation and testing phase, animals were singly housed and placed on a restricted diet of 18g of rat chow per day per rat, with *ad libitum* access to water. Food restriction was aimed at reducing them to 80% of their free-feeding bodyweight and increasing motivation to perform food-reward tasks. A total of 40 adult male Sprague-Dawley rats (6–8 animals/group; 275–300 g from Charles River, Wilmington, MA) were used for these experiments. A separate cohort of animals (n=6) was used for blood glucose measurements to confirm the level of hypoglycemia produced by insulin injections.

### Apparatus

The Set-Shift maze was a rotating 4-arm plus maze with a food well (1.9 cm in diameter and 0.63cm deep) at the end of each arm; arms were 14cm wide × 40.6cm long × 20.3cm high and constructed from painted Plexiglas. The food well was sufficiently deep to hide the 45mg food pellet (Dustless Precision Pellets, purified formula, 45mg; BioServ, Frenchtown, NJ) from the view of the rat. Maze arms varied along each of two stimulus dimensions (brightness and texture), giving arms that were light-smooth, light-rough, dark-smooth, and dark-rough. There was also a holding chamber, 35.6cm × 35.6cm × 35.6cm, constructed of Plexiglas, placed adjacent to the maze that allowed the animals to be placed in it as the maze was being rotated in between trials.

For microdialysis, the table which held the maze and the holding chamber also held an infusion pump (CMA Microdialysis) and a two-channel swivel (Instech Solomon).

### Handling

Animals were extensively handled for a week prior to starting habituation, with the contact time increasing to a minimum of 10 min/animal/day by the end of the week. Food reward pellets were given to the animals in their home cages, after handling, to familiarize them with their taste.

### Set-Shift Maze Habituation

Set-Shift Maze Habituation followed the protocol of Stefani and colleagues for habituating the animals to the Set-Shift maze and for behavioral testing [29, 30, 32]. Briefly, during the open-arm habituation, which lasted for 5 days, animals were familiarized to all 4-arms of the maze and learned to eat reward pellets from the food wells. During closed arm habituation of 5 days animals were placed in the maze when in a T-shape configuration: one arm was blocked at a time and the rat placed in the opposite arm, hence being placed into the stem of a T-configuration. Rats were then allowed to explore whichever arm they chose to enter after leaving the stem, including eating the food reward if it was a pseudorandomly baited arm. Between trials, animals were placed in the holding chamber or held in hands. Inter-trial interval (ITI) was about 22 sec.

### Surgery

Following closed-arm habituation microdialysis probes (CMA12, CMA/Microdialysis) were stereotaxically implanted in the mPFC of the animals anesthetized with 2% isoflurane-oxygen mix (4.2mm anterior to bregma, 0.6mm lateral, and 2.1mm ventral from dura), using standard aseptic surgical technique [33]. Animals were allowed to recover and handled for a week post-surgery.

### RH Induction

RH was induced, in animals randomly assigned to that condition, by i.p. insulin (Humulin, Eli Lilly) administration on each of three consecutive days (doses of 10, 8, and 6 IU/kg respectively), following an established protocol [14]. RH-treated animals were tested on the two days immediately following RH, with normal glycemic levels at the time of testing.

Insulin doses were adjusted to take account of increased sensitivity to repeated insulin treatments, based on previous work and their effect was confirmed in a separate cohort of animals that were not included in the set-shift protocol [14, 15]. Briefly, handled and habituated animals were treated according to the RH protocol and 1 hour post-insulin injection, tail-vein blood was sampled to measure glucose levels using a blood glucose meter (One Touch Ultra Mini).

### Set-Shift Task

Set-Shift testing consisted of two sessions, separated by a 24 hr interval. On Day 1 the animal learned an association between food-reward and one environmental cue (for instance, smooth texture or light color); the remaining two dimensions of arm choice (spatial relation to the start arm and whichever of texture and color is not being linked to reward) offered no information as to the correct arm choice. Learning of this relationship allowed the rat to select the correct arm in order to receive the food reward available on each trial.

On each trial, the rat was placed into a start arm chosen pseudo-randomly with the opposing arm blocked in the T-configuration. Thus, the rat had two possible choices for arms to enter on leaving the initial start arm, and after making a choice and entering the arm the rat was allowed to explore, consume a pellet if present, and then returned to the holding chamber while the maze was prepared (rotated, block moved, cleaned, re-baited as needed) for the next trial. Testing continued until criterion performance level was reached (8 consecutive correct arm choices of the baited arms). Each rat was then tested again, 24h later, with the food-reward contingency changed from a textural to a color variable or vice versa. Testing again continued until the criterion performance level was reached. Day 2 testing required the rat to shift strategy: inhibiting use of the relationship that was previously learned while acquiring and acting on a new, orthogonal contingency relationship. In addition to recording number of trials to criterion on each day, the average latency of each animal to select an arm was recorded as a measure of motor activity. After maze testing animals were sacrificed, brains removed and visually inspected for correct cannula placement. Only data from animals with correctly placed probes were analyzed.

### Microdialysis Procedures

Brain extracellular fluid (ECF) samples were collected in 20-min bins from mPFC using probes with a 2mm long dialysis membrane before, during, and after the behavior testing using previously established protocols [34]. Samples were analyzed for glucose, pyruvate, lactate, and glutamate using a CMA600 analyzer. Dopamine was measured *via* HPLC (ESA, Acton, MA).

### Immunohistochemistry (IHC)

Brains from an additional cohort of animals (n= 6–7/group) treated to induce RH and tested for behavior only (to avoid any confound from the presence of a microdialysis cannula and probe) were processed for chromogen IHC for c-Fos and GluT3 expression using an adaptation of previously published protocols specific for nuclear and membrane protein staining [17, 35, 36]. c-Fos stained nuclei were counted and mean intensity of GluT3 staining in mPFC was calculated.

### Data Analysis

Behavioral data was analyzed using t-test. Biochemical data for glucose, lactate, pyruvate, glutamate, and dopamine were expressed as % of baseline and analyzed as two-way ANOVA (Treatment x Stage of testing) with Student-Newman’s-Keul’s test for post hoc comparisions. The α-level for all statistical comparisions was ≤ 0.05.

## RESULTS

### Efficacy of Hypoglycemia Treatment

No animal experienced coma or seizure. Baseline blood glucose before insulin administration was 137.5 ± 2.5 mg/dl. Insulin administration lowered tail-vein blood glucose to 35.33 ± 6.7 mg/dl; hence, core plasma glucose level (approximately 10 mg/dL higher than that measured from tail vein samples) was approximately 45 mg/dL, in line with our targeted hypoglycemia range [37].

### Set-Shift Performance

**Fig. 1:**
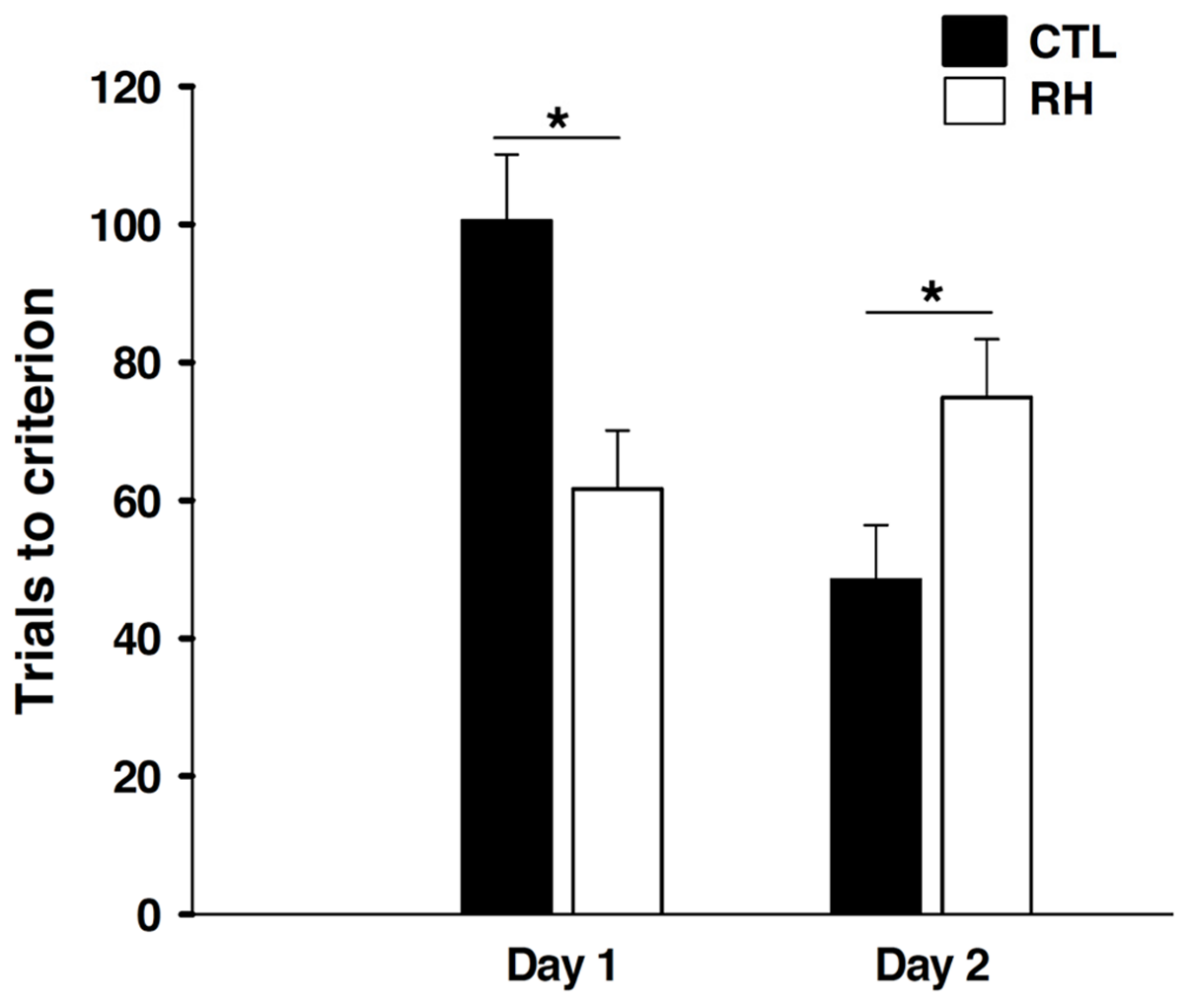
Behavioral performance on the Set-Shift task on day 1 and day 2 of testing in control animals (CTL) and in animals induced with recurrent bouts of hypoglycemia (RH) on each of preceding 3 days of testing. A previous history of recurrent hypoglycemia aided acquisition learning on day 1, however impaired mental flexibility, tested on day 2 in RH group. Data are means + SEM. Asterisks indicate significantly different behavioral performance between the treatment groups (p<0.05).

Fig. (1) shows animals’ performance on both Day 1 (spatial learning) and Day 2 (set-shifting ability) on the Set-Shift task. On Day 1, RH animals learned the task significantly faster than controls [t (14) =3.04; p<0.05], suggesting that antecedent hypoglycemia improved subsequent spatial learning. This finding is consistent with our previous work, which also showed enhanced hippocampal-dependent spatial memory following RH (when tested at euglycemia) [14, 38]. In contrast, on Day 2 RH animals required significantly more trials to reach criterion [t (14) = −2.25; p<0.05], suggesting an impairment in set-shifting performance.

In a separate experiment, we tested whether the effects of RH would interact with glycemic state at the time of testing. Animals being made hypoglycemic on both test days either as their first experience of hypoglycemia (Hypo group) or following previous RH (RH-Hypo group). The rationale for these groups was our previous finding of a significant interaction between RH and acute glycemic state in modulating some cognitive and metabolic processes, including spatial working memory [14, 38]. Not surprisingly, acute hypoglycemia has been shown to impair performance on a variety of cognitive measures [39–45]. However, on this food-rewarded task, both acutely hypoglycemic groups had performance comparable to that of euglycemic control animals, and this appeared to be due at least in part to a confounding effect of hunger on motivation and task performance, so these data were not analyzed further (data not shown).

### Latency

RH and control animals did not differ in average latency to enter choice arms, a proxy measure to control for any differences in motor activity or motivation, on either day of testing (data not shown).

### mPFC Neurochemistry

#### Glucose (Fig. 2)

There were no group differences in the mPFC ECF glucose levels on day 1 of testing. However, on day 2 of testing, mPFC glucose levels of RH animals were elevated compared to controls (F_(1, 69)_ = 6.21, p<0.05).

#### Pyruvate (Fig. 3)

In contrast to glucose, ECF pyruvate levels on day 1 of testing were significantly higher in RH animals than in controls (F_(1, 53)_ = 4.17, p<0.05), while on day 2, RH animals had significantly lower mPFC ECF pyruvate (F_(1, 54)_ = 6.16, p<0.05).

#### Lactate, Glutamate, and Dopamine

ECF lactate and glutamate levels did not vary between the treatment groups on either day of testing. Surprisingly, we saw no changes in mPFC ECF dopamine levels across treatment groups or across stages of testing on either day (data for lactate, glutamate, and dopamine not shown).

### Protein Expression in mPFC

**Fig. 4:**
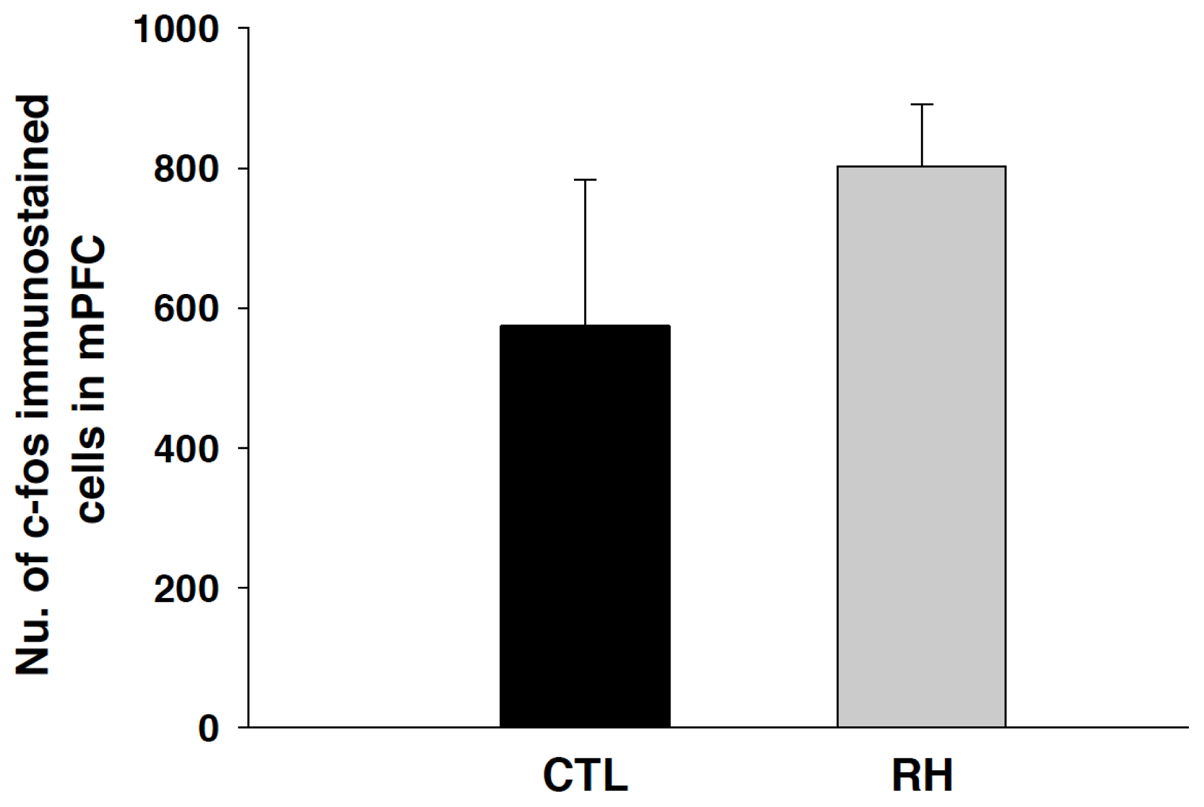

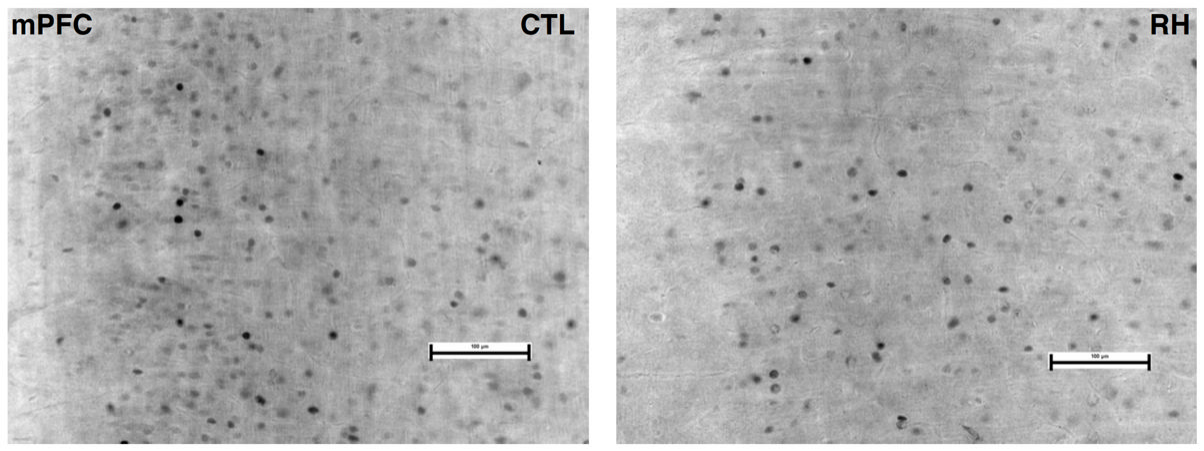
**Fig. (4A).** The mean (+ SEM) number of stained nuclei for c-Fos protein within the medial prefrontal cortex (mPFC) at the end of Set-Shift testing in control animals (CTL) and in animals induced with recurrent bouts of hypoglycemia on each of preceding 3 days of testing (RH). No significant differences were observed in the number of c-fos stained cells between the treatment groups. **Fig. (4B).** A representative medial prefrontal cortex (mPFC) c-Fos immunostaining from CTL and RH animals. Bar = 100μm.

**Fig. 5:**
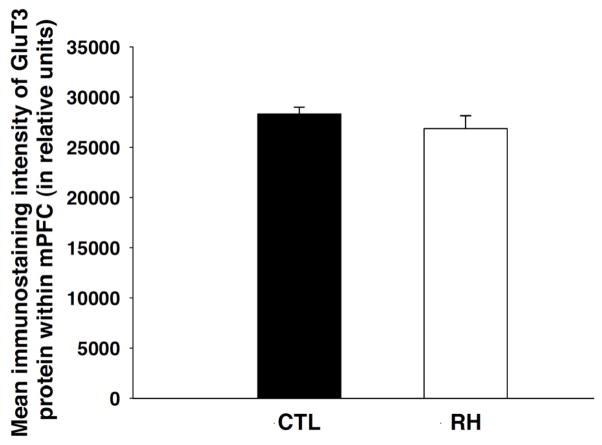

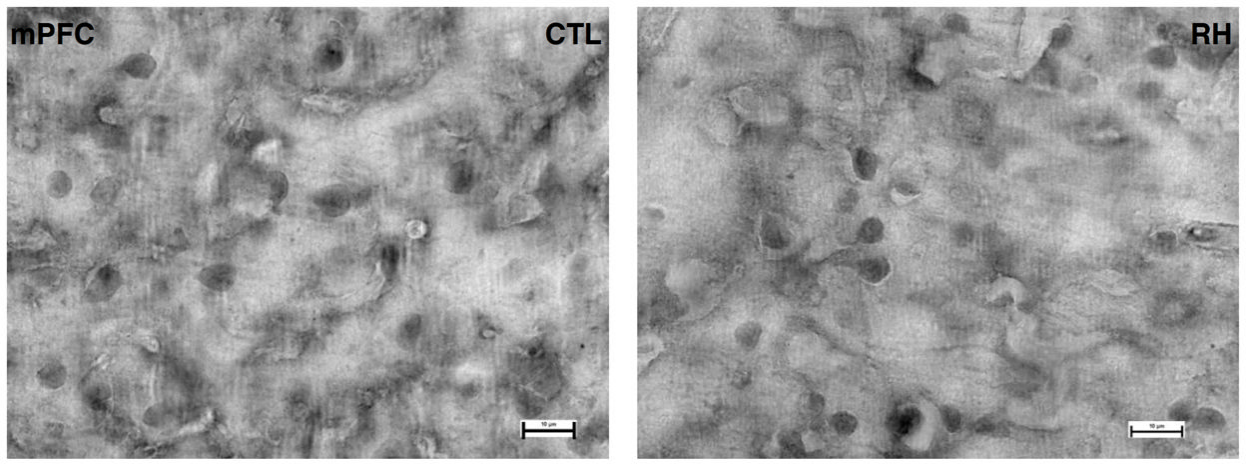
**Fig. (5A).** The mean (+ SEM) staining intensity of glucose transporter 3 (GluT3) immunoreactivity (quantified by thresholded pixels) within the medial prefrontal cortex (mPFC) at the end of Set-Shift testing in control animals (CTL) and in animals induced with recurrent bouts of hypoglycemia on each of preceding 3 days of testing (RH). No significant differences were observed in the mean staining intensity of GluT3 between the treatment groups. **Fig. (5B).** A representative medial prefrontal cortex (mPFC) GluT3 immunostaining from CTL and RH animals. Bar = 10μm.

Prior history of RH but subsequent euglycemia (at the time of testing) did not affect c-Fos expression within the mPFC. Immunolabeling for c-Fos in RH group was comparable to the controls (Fig. 4A). We also saw no difference in mPFC GluT3 expression between groups (Fig. 5A). Representative immunostaining images for c-Fos and GluT3 are depicted in Fig. (4B) and Fig. (5B), respectively.

## DISCUSSION

These data, taken together with previous studies of RH and hippocampal function, present a more complete picture of the neurobehavioral effects of RH. Findings from our studies were consistent with clinical observations. Thus, prior history of hypoglycemia impaired subsequent executive function as demonstrated by impaired set-shifting performance when the rule was changed. However, prior history of hypoglycemia may in fact enhance initial acquisition learning. As the animals learned a contingency rule (day one of testing), animals with a history of RH had increased ECF pyruvate during testing without any difference in ECF glucose, and showed superior learning. This suggests that, again consistent with previous studies brain glucose supply and metabolism may be enhanced as an adaptive response to RH and hence underlie improved cognitive performance [14, 15]. In contrast, on day two of testing, when the contingency rule was altered and demand on the mPFC would have been expected to be highest, RH animals had higher mPFC ECF glucose and lower pyruvate levels. These data suggest that less glucose was being metabolized within the mPFC, which may impair behavior. This explanation is also consistent with the idea that altered glucose metabolism may underlie some of cognitive effects of RH. Conversely, post-mortem mPFC c-Fos expression was not significantly altered by RH. This finding suggests that at the point at which performance criterion was reached, total mPFC activity may have been similar across groups, but that the RH animals achieved that state more slowly (requiring more trials), once again indicative of a lower level of metabolism. Given that we saw no alteration in mPFC GluT3 following RH, glucose supply to neurons is unlikely to be significantly different between groups. This suggests that the behavioral impairment observed in our study may be due to changes in glucose demand rather than being affected by glucose delivery to the mPFC. It is known that in response to pathophysiological metabolic changes, free radicals like NO can regulate GluT-3-mediated glucose uptake and trigger glycolysis independent of changes in membrane-protein expression [46]. Although this possibility was not tested in the present study, the fact that we observed significant group differences supports the suggestion that the impact of RH on glucose metabolism is sufficient to overcome such compensatory mechanisms. However, an effect of RH to increase other glucose transporters (e.g. GluT1 at the blood-brain barrier) cannot be ruled out, and studies have reported upregulation in whole-brain or cortical GluT1 expression consequent to chronic hypoglycemia [47, 48]. Overall, the elevated mPFC metabolic activity observed on Day 1 suggests that the requirement for learning of an association between a particular maze attribute and reward places significant load on the mPFC, consistent with literature findings [22, 23, 31, 49, 50].

The results are consistent with, but significantly extend, past findings. McNay & Sherwin showed, using an identical short-term RH model, that both, performance on a hippocampally-mediated spatial task and hippocampal metabolism were enhanced following RH when tested at euglycemia but *impaired* when measured during subsequent hypoglycemia [14]. Importantly, the effects of RH were identical in diabetic and control animals at behavioral, neural, and metabolic levels, as well as with subsequent long-term studies of RH in rodents, strongly supporting the use (as here) of non-diabetic animals in the study of RH in order to avoid any potentially confounding comorbidities of diabetes such as neuropathy, vision impairment, and so on [15]. The present results from Day 1 are highly consistent with the euglycemic portion of those findings; the key finding from the present experiment is that when the task demands are altered to require cognitive shifts and inhibition of prior learning, antecedent RH *impairs*, rather than enhances, performance, even at euglycemia. Thus, it appears that the impact of RH on subsequent cognitive and neural performance varies by brain region and the details of cognitive demand.

The RH-induced behavioral impairment on the Set-Shift task could not be explained by an alteration in mPFC dopamine release. Dopaminergic neurotransmission is known to play an important role in mediating mental flexibility [26, 51–53]; mPFC dopamine release is not critical for rule acquisition on day 1 of testing but is more important for shifting strategies [22, 30]. However, as we did not find a significant RH treatment effect on mPFC dopamine on either days of testing, our data suggest that some other neurochemical system may underlie the effects of RH on set-shift performance, rather than dopamine. Consistent with this, one previous study has suggested that dopamine release (albeit under different conditions) is unaffected by alterations in brain glucose metabolism that persist for up to 80 minutes [54].

Our data show that a history of prior hypoglycemia may lead to impaired set-shifting ability, which is a measure of decision-making ability, consistent with clinical reports of impaired mental flexibility in patients with diabetes. Moreover, the behavioral impairment is associated with reduced mPFC glucose metabolism subsequent to RH, despite systemic euglycemia when tested. These data have important implications for diabetics undergoing insulin replacement therapy: especially when taken together with previous findings, they strongly suggest that mental function is altered following even short-term, moderate RH. Further research may be necessary to develop treatment regimens which reduce the risk of RH and at the same time achieve optimal glycemic control.

## Figures and Tables

**Fig. 2:**
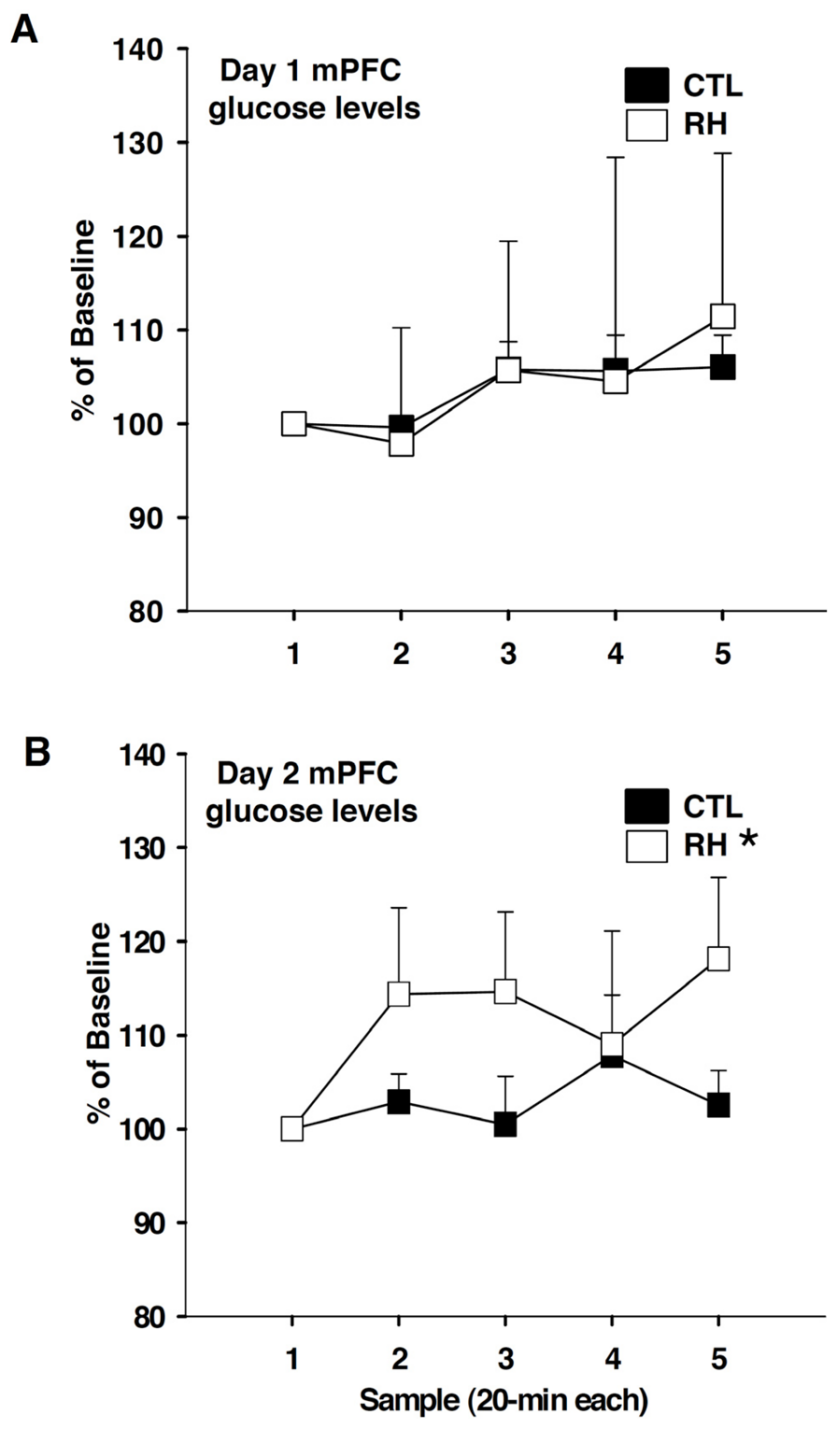
Medial prefrontal cortex (mPFC) extracellular fluid (ECF) glucose levels, measured before, during, and after Set-Shift testing of control animals (CTL) and animals with prior history of recurrent hypoglycemia (RH) on day 1 (A) and day 2 (B) of the behavior task. ECF samples were collected in 20 min bins. Data are expressed as percentage of baseline + SEM. Asterisks indicate significantly different group findings (p<0.05).

**Fig. 3:**
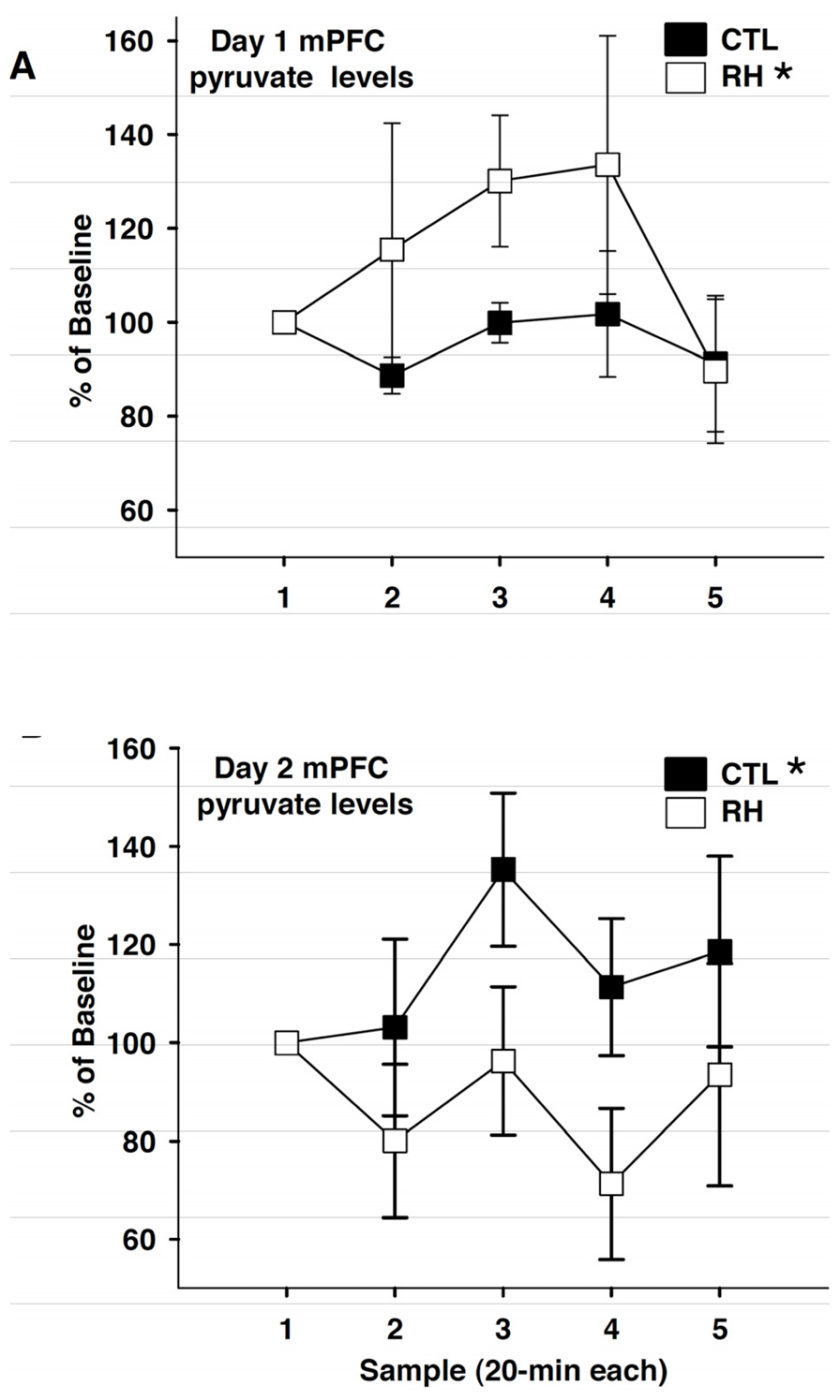
Medial prefrontal cortex (mPFC) extracellular fluid (ECF) pyruvate levels, measured before, during, and after Set-Shift testing of control animals (CTL) and animals with prior history of recurrent hypoglycemia (RH) on day 1 (**A**) and day 2 (**B**) of the behavior task. ECF samples were collected in 20 min bins. Data are expressed as percentage of baseline + SEM. Asterisks indicate significantly different group findings (p<0.05).
